# Adapt or perish

**DOI:** 10.7554/eLife.83617

**Published:** 2022-11-08

**Authors:** Rohan BH Williams

**Affiliations:** 1 Singapore Centre for Environmental Life Sciences Engineering, National University of Singapore Singapore Singapore

**Keywords:** microbiome, antimicrobial resistance, metagenomics, wastewater treatment, None

## Abstract

Microbial communities in wastewater treatment plants provide insights into the development and mechanisms of antimicrobial resistance.

**Related research article** de Nies L, Busi SB, Kunath BJ, May P, Wilmes P. 2022. Mobilome-driven segregation of the resistome in biological wastewater treatment. *eLife* **11**:e81196. doi: 10.7554/eLife.81196.

Bacteria and other microbes are probably the most successful life forms on Earth. They are ubiquitous and can survive in a large range of habitats, from extreme environments to the human body. One of the reasons for their success is their potential to adapt to changing conditions, including drug treatments.

Antimicrobial resistance – the ability of bacteria to evolve resistance to drug treatments, including antibiotics – poses a major threat to health interventions (McEwen and Collignon, 2018). Although antimicrobial resistance occurs naturally, the widespread and often uncontrolled use of antibiotics in both humans and livestock have exacerbated this ability. As a consequence, treatment of many common bacterial infections, such as sepsis, or urinary and sexually transmitted infections, is now compromised.

Bacteria acquire antimicrobial resistance in two main ways: one is through favourable mutations in the DNA during cell replication; the other is by exchanging genetic material that often contain genes mediating antimicrobial resistance, also known as horizontal gene transfer (Haudiquet et al., 2022). While the mechanisms of genetic transfers – and how they contribute to antibiotics resistance – have been understood for decades, it has been less clear how they work in the ‘real world’. Now, in eLife, Paul Wilmes and colleagues at the University of Luxembourg – including Laura de Nies as first author – report new insights about antimicrobial resistance using wastewater treatment plants as an example (de Nies et al., 2022).

While microbial communities in wastewater thrive on the nutrient-rich streams from sewage systems, they also encounter a range of micropollutants arising from human domestic and industrial activity, including antibiotics. Bacteria – including the ones carrying antimicrobial resistance genes – also enter the wastewater system. These conditions provide ample opportunities for the evolution and/or transmission of antimicrobial resistance. Subsequently, the risk of such resistant bacteria being transmitted into natural water systems and eventually back into human or animal populations, is extremely high (Pruden et al., 2021).

The researchers analysed previously collected multi-omics datasets that contained sequences of all the DNA found in a wastewater treatment plant. This allowed them to identify both the genomes of species within the community and the mobile genetic elements that can be transferred between bacteria. Using metatranscriptomics and metaproteomics, two techniques that measure which genes are active (McDaniel et al., 2021), de Nies et al. were able to further analyse gene expression at the level of the entire microbial community. Samples were collected over one and a half years, which allowed the researchers to assess the dynamic changes in the inter-relationship between microbes and mobile genetic elements.

In total, de Nies et al. identified 29 different major types of antimicrobial resistance genes. The relative abundance of these types changed slightly over time, which could be linked to changes in resistant entities within the community, either due to the transfer of such genes, to changes in the composition of bacteria, or both. Overall, antimicrobial genes that provide protection against multiple drugs, and those that provide resistance against two common types of antibiotics (aminoglycosides and beta lactams) were both abundant and highly expressed.

Interestingly, the most highly expressed genes were related to resistance against antimicrobial peptides, which are part of the innate immune system in multicellular organisms. One of these was a gene called YojI, which encodes resistance to microcin, a common toxin that is widely produced by bacteria and other prokaryotic species. It was found in about 90% of all expressed transcripts attributed to this type of antimicrobial resistance, suggesting that many species in the community produce microcin as a survival strategy, and thus also require resistance to toxins produced by other species.

To better understand the mechanisms underlying antimicrobial resistance within microbial communities in wastewater, de Nies et al. next focused on two relevant types of mobile genetic elements that convey antimicrobial resistance genes: plasmids (small, circular DNA molecules) and bacteriophages (viruses that infect bacteria). The analyses confirmed that the majority of antimicrobial resistance genes are harboured in bacterial chromosomal DNA, but plasmids and phages nevertheless transmitted 11% and 7% of those genes, respectively.

There appears to be a preferential link between the types of resistance genes and the types of mobile genetic element that carry them. Further analyses indicated that several human pathogenic bacteria only express antimicrobial resistance genes associated with plasmids, which suggests that these genes may be more easily and widely transmitted. The study by de Nies et al. also documents a wide variety of resistance genes in a key set of human pathogens, known as the ESKAPEE species, that are also present in the microbial community of the wastewater treatment plant.

The work of de Nies et al. highlights how variable the transmission of resistance pathways within complex environments can be. More targeted observational studies may be warranted to fully understand the transmission flows of these genetic materials. For example, the new metagenomic assays that can infer the colocalization of DNA from chromosomes and mobile genetic elements (within the same microbial cell) would paint a more accurate picture (Stalder et al., 2019); but these techniques are also much more complex compared to bulk DNA sequencing.

Wastewater treatment plants play a critical role in both mitigating the impact of human waste on natural water sources and preventing ‘feed-backs’ of pathogens into human populations. They are also important surveillance systems that can monitor the spread of viruses that people shed in their faeces. Understanding the various pathways of resistance transmission – including the role of plasmids and phages – will help to understand the ecological relationships between human, animals and the natural environment. In the future, wastewater plants could be used to monitor antimicrobial resistance and their potential threat to human health, and to guide initiatives that prevent the release of such resistant bacteria back into the environment (Pruden et al., 2021).
